# Editorial: Umbilical cord blood and tissue in novel therapies and haematopoiesis research

**DOI:** 10.3389/fcell.2022.979306

**Published:** 2022-08-09

**Authors:** Diana Hernandez, Robert David Danby, Sergio Querol

**Affiliations:** ^1^ Anthony Nolan Research Institute, London, United Kingdom; ^2^ UCL Cancer Institute, University College London, London, United Kingdom; ^3^ Oxford University Hospitals NHS Trust, Oxford, United Kingdom; ^4^ Banc de Sang i Teixits, Barcelona, Spain

**Keywords:** umbilical cord blood, cell and gene therapies, cord blood banking, iPSC (induced pluripotent stem cell), Autism Spectrum Disorder ASD, cord blood transplantation (CBT)

Umbilical cord blood (UCB) was originally identified as a rich source of hematopoietic stem cells, and an alternative to bone marrow for hematopoietic cell transplantation (HCT) in 1988. Since then, it has been used extensively for this purpose, but it has also emerged as an important source of hematopoietic cells for the study of human hemopoiesis, and the development of novel therapy treatments. In an era of massive change in treatment modalities, many based on adoptive cell therapies, finding sources of cells which pose no harm to the donor and are readily available has increased. This had therefore fuelled the interest in understanding the biology of cord blood cells and how they can be best utilized for the manufacture of off the shelf therapies for the treatment of both haematological and non-haematological diseases.

This issue highlights three very important aspects of cord blood and tissue: (1) Why is UCB still a crucial source of cells for HCT? (2) How can novel *ex vivo/in vivo* manipulation be used to overcome some of disadvantages of UCB? and (3) How do we make the most of the resources already available to maintain UCB banking, to increase the utilisation of existing inventory, and have less wastage of collected units?

In this topic issue, Wynn et al. discuss the importance of UCB as a source of HCT, the advantages it offers specifically in the paediatric setting where it is used for treatment of both malignant and non-malignant conditions, importantly metabolic and immunodeficiency disorders. Despite the recent worldwide decline in the use of UCB, the authors argue for the continued support of the banking effort as it offers very specific advantages in the paediatric setting where urgency in transplant means the need for readily available grafts is crucial.

Milano et al. go on to explore the use of *ex-vivo* expanded UCB cells as an adjuvant therapy to increase the chances of success of conventional UCB transplant by providing haematopoietic progenitors which improve time to neutrophil engraftment. Crucially, these cells do not appear to persist for more than 2 weeks, which means they are less likely to have unwanted side effects such as graft-versus-host disease.

Additionally, Tamouza et al. discuss the use of UCB and UC derived MSCs for the treatment of non-haematological disease, specifically autism spectrum disorders (ASD), as an example of neurological disorders that may have an autoimmune component. With advances in conditioning regimens, which may have previously been regarded as too high risk for the treatment of non-life-threatening diseases, HCT can now be offered. These early findings show signs of some efficacy, though more studies are needed as well as better understanding of the mechanisms of action.

However, in order to be able to use UCB for conventional and novel treatments, the sustainability of CB banking is crucial. This argument is made eloquently by Rebulla et al., who make calculations around the number of units needed in order to serve the population they were created for versus the cost of banking UCB units that may go unutilised. They highlight the importance of diversification and maximising the utilisation of the collected units. The authors specifically discuss the arrangements made in Italy and Spain to facilitate the collection of plasma components for immediate use in patients but also for future manufacture of plasma containing products, such as eye drops. This diversification may provide a much-needed source of revenue for public CB banks, struggling with lower rates of use of cord in the HCT setting. Moreover, we have seen in recent years the increased interest in the use of cord blood cells as starting materials for the production of immunotherapy products such as NK-CARs, but also as starting materials for the creation of iPSC lines with particular HLA haplotypes. A readily reproducible methodology to do this is presented by Tian et al. in this issue, rounding up the multifaceted usages of UCB for current and future therapies.

It is important to highlight that the collection of UCB can be emotive and controversial, and that it is essential that the collection, manipulation and use of this precious material is carried out with the strictest and highest ethical and safety standards to ensure the continued support of donors and the general public. Adherence to high quality standards is also necessary to reassure users that the product they are requesting has the expected characteristics and will perform accordingly. With effective safeguards in place and appropriate infrastructure to deliver diversification in cord usage, we can ensure that high quality UCB banks can not only survive but thrive. Thus, ensuring continuity of supply for patients in need of HCT, but also for novel treatment modalities, especially in the setting of Cell and Gene Therapies.

