# Erratum: Editorial: Umbilical cord blood and tissue in novel therapies and haematopoiesis research

**DOI:** 10.3389/fcell.2022.1066382

**Published:** 2022-10-24

**Authors:** Frontiers Production Office

**Affiliations:** Frontiers Media SA, Lausanne, Switzerland

**Keywords:** umbilical cord blood, cell and gene therapies, cord blood banking, iPSC (induced pluripotent stem cell), autism spectrum disorder ASD, cord blood transplantation (CBT)

Due to a publishing error the first two affiliations were listed incorrectly. Instead of “UCL Cancer Institute, Hampstead, United Kingdom”; and “University College London, London, United Kingdom,” they should be as follows:

“1 Anthony Nolan Research Institute, London, United Kingdom

2 UCL Cancer Institute, University College London, London, United Kingdom”

The publisher apologizes for this error and states that this does not change the scientific conclusions of the article in any way. The original article has been updated.

